# Adrenomedullin blockade improves catecholamine responsiveness and kidney function in resuscitated murine septic shock

**DOI:** 10.1186/cc10629

**Published:** 2012-03-20

**Authors:** K Wagner, U Wachter, J Vogt, S Weber, M Groeger, O McCook, M Georgieff, A Bergmann, H Luettgen, E Calzia, P Radermacher, F Wagner

**Affiliations:** 1 University Medical School Ulm, Germany; 2 AdrenoMed AG, Henningsdorf, Germany

## Introduction

The effects of adrenomedullin in circulatory shock states are controversially discussed: while its exogenous supplementation improved organ function and survival [1] in experimental models due to maintenance of hyperdynamic hemodynamics [2] in otherwise hypodynamic conditions, high blood levels were associated with increased mortality in patients with septic shock [3], most likely as a result of excessive vasodilatation [4] and/or impaired systolic heart function [5].

## Methods

Immediately after cecal ligation and puncture to induce peritonitis, mice randomly received vehicle (*n *= 11) or the adrenomedullin antibody HAM1101 (*n *= 9; 2 μg/g to achieve antibody concentrations >4 ng/ml). Fifteen hours later animals were anesthetized, mechanically ventilated and instrumented for a consecutive 6-hour observation period. Colloid fluid resuscitation and continuous i.v. noradrenaline were titrated to maintain normotensive (mean blood pressure >60 mmHg) and hyperdynamic hemodynamics. Creatinine blood levels and clearance were assessed as surrogate for glomerular filtration [6,7]. All data are median (quartiles).

## Results

Adrenomedullin antagonism decreased the noradrenaline requirements needed to achieve target hemodynamics (0.009 (0.009; 0.012) vs. 0.02 (0.015; 0.044) μg/g/hour, *P *< 0.001), increased total diuresis (2.6 (2.3; 3.9) vs. 0.6 (0.5; 2.7) ml, *P *= 0.028) resulting in improved fluid balance (0.18 (0.14; 0.2) vs. 0.26 (0.19; 0.27), *P *= 0.011) and kidney function (creatinine levels at the end of the experiment: 1.3 (1.2; 1.5) vs. 2.0 (1.5; 2.9) μg/ml, *P *= 0.006; creatinine clearance: 400 (316; 509) vs. 197 (110; 301) μl/minute, *P *= 0.006).

## Conclusion

In resuscitated murine septic shock, early modulation of excess adrenomedullin activity via antibody HAM1101 improves cardiovascular catecholamine responsiveness, ultimately associated with attenuation of acute kidney injury.
